# A RADIO-BASED NONCONTACT MONITORING SYSTEM FOR NIGHTTIME VITAL SIGNS AND SLEEP INSIGHTS

**DOI:** 10.1093/geroni/igae098.1543

**Published:** 2024-12-31

**Authors:** Leo He, Yindong Hua, Mengjing Liu, Zongxing Xie, Elinor Schoenfeld, Fan Ye

**Affiliations:** Stony Brook University, Stony Brook, New York, United States; Stony Brook University, Stony Brook, New York, United States; Stony Brook University, Stony Brook, New York, United States; Stony Brook University, Stony Brook, New York, United States; Stony Brook University School of Medicine, Stony Brook, New York, United States; Stony Brook University, Stony Brook, New York, United States

## Abstract

A passive method for daily sleep monitoring offers critical insights into health and well-being as variations in sleep patterns and vital signs can indicate the onset or progression of health issues (e.g., stroke, dementia, sleep apnea). Existing technologies are mostly designed for clinical purposes (e.g., polysomnography machines), or impose physical and/or cognitive burdens (e.g., smart watches), rendering them unsuitable for longitudinal, home-based monitoring. We have developed a non-contact, radio-based monitoring system. Our approach utilizes ultra- wideband radio sensors, coupled with sophisticated Region-of-Interest localization (the area of strongest displacement caused by vital signs), vital sign extraction (weighted estimators from the intermodulation and harmonic phase data), and analysis algorithms (body movement removal and sleep stage classification). Our passive, cost-effective, home-based monitoring is free of physical and cognitive burdens, suitable for older adults with physical and/or cognitive limitations and limited expendable income. Our algorithms can identify the trends of vital signs, and offer preliminary differentiation between deep, REM, light sleep, and awake time throughout the night based on our pilot 59 hours’ sleep data from 6 subjects. By enabling long-term, continuous observation of sleep patterns and vital signs without any physical interaction, the insights gained from our system hold significant potential for early detection and intervention in health issues, paving the way for a higher quality of life for older adults. To be presented are the methods used to collect these data, results and interpretation of our findings, and the role that such data can play to help with aging in place.

